# Time Course of Changes in Peripheral Blood Gene Expression During Medication Treatment for Major Depressive Disorder

**DOI:** 10.3389/fgene.2019.00870

**Published:** 2019-09-18

**Authors:** Ian A. Cook, Eliza Congdon, David E. Krantz, Aimee M. Hunter, Giovanni Coppola, Steven P. Hamilton, Andrew F. Leuchter

**Affiliations:** ^1^Neuromodulation Division, Semel Institute for Neuroscience and Human Behavior, University of California, Los Angeles, Los Angeles, CA, United States; ^2^Department of Psychiatry & Biobehavioral Sciences, David Geffen School of Medicine, University of California, Los Angeles, Los Angeles, CA, United States; ^3^Department of Bioengineering, Henry Samueli School of Engineering at Applied Science, University of California, Los Angeles, Los Angeles, CA, United States; ^4^Department of Psychiatry, Kaiser Permanente Northern California, San Francisco, CA, United States; ^5^Department of Psychiatry, University of California, San Francisco, San Francisco, CA, United States

**Keywords:** biomarkers, predictive markers, neuroscience, gene expression, antidepressant treatment

## Abstract

Changes in gene expression (GE) during antidepressant treatment may increase understanding of the action of antidepressant medications and serve as biomarkers of efficacy. GE changes in peripheral blood are desirable because they can be assessed easily on multiple occasions during treatment. We report here on GE changes in 68 individuals who were treated for 8 weeks with either escitalopram alone, or escitalopram followed by bupropion. GE changes were assessed after 1, 2, and 8 weeks of treatment, with significant changes observed in 156, 121, and 585 peripheral blood gene transcripts, respectively. Thirty-one transcript changes were shared between the 1- and 8-week time points (seven upregulated, 24 downregulated). Differences were detected between the escitalopram- and bupropion-treated subjects, although there was no significant association between GE changes and clinical outcome. A subset of 18 genes overlapped with those previously identified as differentially expressed in subjects with MDD compared with healthy control subjects. There was statistically significant overlap between genes differentially expressed in the current and previous studies, with 10 genes overlapping in at least two previous studies. There was no enrichment for genes overexpressed in nervous system cell types, but there was a trend toward enrichment for genes in the WNT/β-catenin pathway in the anterior thalamus; three genes in this pathway showed differential expression in the present and in three previous studies. Our dataset and other similar studies will provide an important source of information about potential biomarkers of recovery and for potential dysregulation of GE in MDD.

## Introduction

The mechanism of action (MOA) of antidepressant medications remains incompletely understood. Antidepressant medications are remarkably pleiotropic in their effects. In addition to binding to serotonin and/or norepinephrine transporters (SERT and NET, respectively), their affinity for other neurotransmitter receptors and ligand-gated ion channels may be relevant to their effects (Bianchi and Botzolakis, 2010). In addition, the acute effect on aminergic signaling has the potential to influence a wide range of downstream genetic pathways implicated in neurogenesis, synaptic plasticity, neuronal excitability, and metabolism (Baudry et al., 2011). To date, the identity of genes that are consistently up- or down-regulated by antidepressants remains obscure. The application of genomic techniques to patients undergoing treatment with antidepressants has the potential to identify clusters of genes and/or pathways that undergo transcriptional regulation in response to these drugs. Identification of reproducible gene expression (GE) changes during antidepressant treatment will be the first step toward the long-term goal of elucidating which changes are required for the relief of depressive symptoms (Bartova et al., 2010; Baudry et al., 2011; Redei et al., 2014).

Because of the limited accessibility of neuronal tissue, studies of the transcriptome in patients undergoing treatment for MDD have focused upon GE changes in peripheral blood (Tylee et al., 2013). Studies often have been of limited size but have examined changes during treatment with a variety of medications (Belzeaux et al., 2010; Mullins et al., 2014; Watanabe et al., 2015; Miyata et al., 2016) or cognitive behavioral therapy (Redei et al., 2014). To our knowledge, no previous study has systematically examined the changes in peripheral blood GE at multiple time points in an 8-week course of antidepressant treatment. We report here a study of the transcriptional changes that occur in response to treatment with escitalopram or bupropion in a cohort of 68 subjects at 1, 2, and 8 weeks of medication treatment, and the association of these changes with clinical outcome.

## Methods

### Subjects

Samples were collected from a subset of the adult subjects (age 21–75) who participated in a double-blind controlled trial conducted to evaluate a neurophysiologic biomarker for use in assigning antidepressant medication treatment (NCT00917059 “Personalized Response Indicators of SSRI Effectiveness in Major Depression” [PRISE-MD]). The objective of the overall trial was to assess outcomes when subjects were prospectively assigned to antidepressant treatment based upon biomarker status, using a quantitative electroencephalographic (qEEG) biomarker that previously had been shown to be predictive of response and remission during treatment with escitalopram (ESC) and bupropion (BUP) (Leuchter et al., 2009a; Leuchter et al., 2009b; Cook et al., 2013). All subjects started with 1 week of single-blind ESC (10 mg each morning) for measuring the biomarker and then were randomized to continue on ESC or switch to BUP (150 mg each morning) under double-blind conditions for the remainder of the protocol. Randomization was performed using a biomarker-based stratification to ensure that biomarker-positive and biomarker-negative subjects received balanced allocation to the treatments. The study tested the hypothesis that treatment concordant with the biomarker would lead to better outcomes than biomarker-discordant assignment. ESC outcomes were assessed after 7 weeks of continuous treatment (study week 7) while BUP outcomes were assessed after 7 weeks of continuous BUP (study week 8). The study was statistically powered to detect a difference for subjects assigned to ESC but not for those receiving BUP. In accordance with principles of the Helsinki Declaration, all protocols were approved by the UCLA Institutional Review Board, and all subjects provided written informed consent.

All subjects were diagnosed with MDD based upon structured interview data (Mini-International Neuropsychiatric Interview, or MINI) (Sheehan et al., 1998) and had a symptom severity score of 12 or greater on the Quick Inventory of Depressive Symptomatology—Self Rated version (QIDS-SR16) (Rush et al., 2003). Subjects were excluded for serious medical or psychiatric comorbidities, recent exposure to electroconvulsive therapy, and other factors that would either pose safety concerns or render data uninterpretable (detailed at clinicaltrials.gov). Subjects were evaluated in an outpatient setting every week for evidence of symptomatic changes and/or side effects. The primary outcome measure was the score on the 17-item Hamilton Depression Rating Scale (Ham-D_17_) (Hamilton, 1960). Degree of improvement was defined as the change in the Ham-D_17_ score from pretreatment baseline to endpoint, with response defined as a decrease in Ham-D_17_ of ≥50% and remission as a final Ham-D_17_ of ≤7.

A total of 274 adults were screened, 180 were enrolled and randomized, and 133 completed at least 4 weeks of the trial and had evaluable clinical data, with usable samples for genomic assays collected from a subset of 68 individuals (40 ESC, 28 BUP) at up to four time points during the trial: pre-treatment baseline and 1, 2, and 8 weeks of treatment in the protocol (see **Table 1** for a breakdown of samples by treatment and time point).

**Table 1:** Number of samples by time point and medication.

	Escitalopram (ESC)	Bupropion (BUP)	*Total*
Baseline	40	24	*64*
Week 1	34	23	*57*
Week 2	35	26	*61*
Week 8	36	28	*64*
*Total*	*145*	*101*	*246*

### RNA Extraction and Measurement of Gene Expression

Whole blood was collected using PAXgene Blood RNA tubes (PreAnalytiX GmbH, Hombrechtikon, Switzerland) and frozen until time of extraction. Samples were extracted in one batch using a PAXgene Blood RNA Kit (Qiagen, Valencia CA); quantity and quality of RNA samples were checked using an Agilent Bioanalyzer 2100 (Agilent, Santa Clara CA) in the UCSF Genome Core Facility. Total mRNA was amplified and labeled using the Illumina TotalPrep RNA Amplification Kit before being hybridized to the Illumina Expression RefSeq HT-12 BeadChip, querying the expression of ∼47,000 RefSeq transcripts, as per manufacturer’s protocol. The BeadChips were then scanned with the Illumina iScan system and signal was extracted using the Illumina BeadStudio Software (Illumina, San Diego, CA).

Illumina expression assays were run on blood samples meeting standard QC criteria. After assay, raw data were inspected to remove samples with missing data for the majority of probes. Data were inspected after log_2_ transformation to screen for outliers, using visual inspection of correlations among samples and probes and calculating z-scores to find samples with low correlations with the rest of the dataset. A total of 14 samples were excluded based on QC of raw data, and the resulting 246 samples were carried forward for analysis.

Raw GE data were preprocessed using Bioconductor packages for R following protocols developed by the UCLA Informatics Center for Neurogenetics and Neurogenomics (ICNN). Expression data were log_2_ transformed and quantile normalized; no batch correction was necessary as all samples were processed at the same time. As multiple probes may tag the same gene on the array, the collapseRows function was used to select a single probe per gene, using the absolute maximum mean expression values of the multiple probes. Quality-control analysis was performed by examining the inter-array Pearson correlations, clustering based on variance, and the mean absolute deviation (MAD) using the top 1,000 most variant probes. Twelve outliers were identified based on GE data and removed from further analyses. After quality control steps were applied, 28,105 gene transcripts were present in 246 samples from 68 unique subjects.

### Data Analysis

Analyses of demographic and clinical outcome data were performed using SPSS (SPSS, Inc.; Chicago, IL). Outcome and demographic differences were compared between outcome groups using Student’s T-test. Analysis of differential GE was performed using a linear model fitting (LIMMA package), accounting for within-subject variance. Age, sex, and RNA integrity number (RIN) were included as covariates in all analyses. After linear model fitting, a Bayesian estimate of differential expression was calculated applying a nominal p-value threshold of 0.005. The resulting gene lists were interrogated using the data-driven tools described below.

### Gene Ontology and Gene Pathways

We used the anRichment function within the WGCNA package in R (Langfelder and Horvath, 2008; Miller et al., 2011) to test for enrichment of: (1) gene ontology (GO) biological process terms and (2) pathway-based categories (e.g., NCBI BioSystems Reactome pathways). Enrichment analyses are conducted using a hypergeometric test, which can be used to identify whether a sub-population (e.g., differentially expressed genes) is over-represented in a given sample (e.g., genes annotated with a specific function), when drawing from the larger population of probes. This test is based on a discrete probability distribution, representing the likelihood of obtaining a number of successful draws, out of N possible draws, without replacement from a finite population. To adjust for multiple comparisons, we applied a local false discovery rate (FDR) correction and report q-value estimates using the R q-value package for all enrichment tests.

### Cell Type–Specific Enrichment Analyses

We queried data available in the RNA-seq database (Zhang et al., 2014) to identify gene sets preferentially enriched in cell types highly represented in the nervous system, including astrocytes, endothelial cells, microglia, neurons, and oligodendrocytes (further subdivided by precursors, newly formed, and myelinated oligodendrocytes). Following the authors’ recommendations, the top 500 enriched genes (using FPKM > 20) were selected for each cell type. Because of evidence that the anterior thalamus may constitute a site for antidepressant medication action, we tested for enrichment of genes preferentially expressed in the thalamic structures (Hawrylycz et al., 2012), as well as genes involved in the Wnt/β-catenin pathway in the anterior thalamus (Wisniewska et al., 2012).

### Reference Gene Sets From Relevant Published Studies

After a comprehensive review of the literature, published studies relevant to the current data set were compiled. This includes studies focusing on GE differences in MDD patients *vs.* controls, or within MDD patients as a function of treatment (Mamdani et al., 2011; Belzeaux et al., 2012; Menke et al., 2012; Liu et al., 2014; Mamdani et al., 2014; Guilloux et al., 2015; Hennings et al., 2015; Hodgson et al., 2016; Jansen et al., 2016). This includes studies of peripheral blood GE changes in humans undergoing treatment with citalopram and psychotherapy (n = 34) (Guilloux et al., 2015), citalopram alone (n = 77) (Mamdani et al., 2014), a variety of different psychotropic agents as well as ECT (n = 16) (Belzeaux et al., 2012), nortriptyline or ESC (n = 136) (Hodgson et al., 2016), or citalopram (n = 34) (Belzeaux et al., 2016). We examined genes sets differentially expressed in these previous studies to determine whether there was significant overlap with the present study, as well as to determine whether individual genes were reported in more than one previous study of antidepressant treatment, as well as the Wnt/β-catenin pathway.

### Drug-Induced Gene Changes in Mice

Using the Genomic Signature Identification tool in the genes2mind database, we extracted lists of genes that represent drug-specific genomic signatures in the mouse brain following antidepressant administration. Specifically, we extracted the top 100 genes associated with BUP or fluoxetine at 1, 2, 4, and 8 hours and all time points.

### Exploratory Pathway Analysis

Ingenuity Pathway Analysis (IPA) was used to explore biological networks enriched by genes identified through the differential expression analysis. Follow-up exploratory analyses were conducted to examine differences as a function of treatment type and response status.

## Results

### Clinical Outcome

Of the 68 subjects, 67.7% responded and 47% remitted with treatment with a mean decrease in HamD_17_ score of 60% (**Table 1**); by treatment group, 60.0% (37.5%) of ESC subjects and 78.6% (60.7%) of BUP subjects responded (remitted), respectively. There was a trend toward a greater proportion of male patients and a greater degree of improvement in the group of patients treated with BUP, although these differences were not statistically significant between the two treatment conditions. Thirty-six of the ESC- and 28 of the BUP-treated subjects completed 8 weeks of treatment. The number of subjects who completed treatment who had blood drawn for GE studies at each time point is shown in **Table 1**.

### Gene Expression Analyses

**Figure 1:**
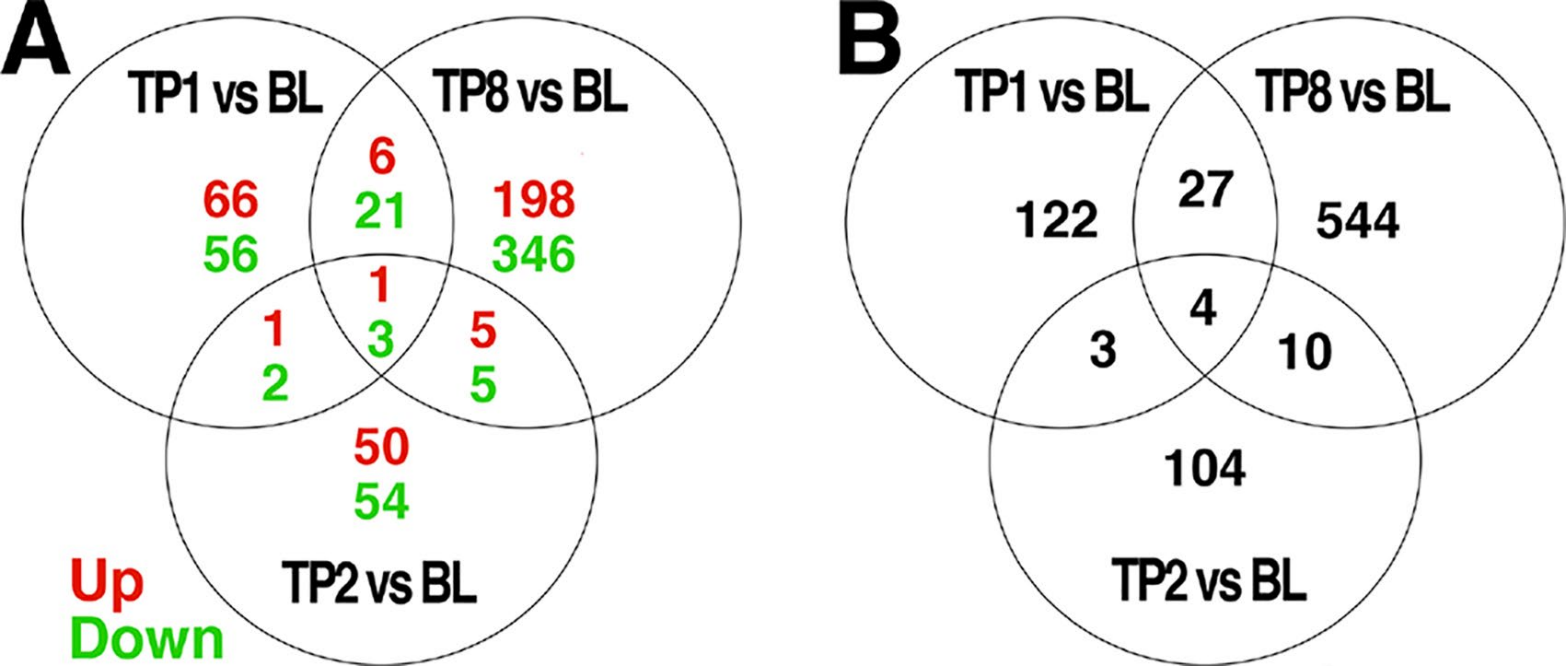
Venn diagrams of genes differentially expressed over time, and the intersection of genes between each comparison. Differential expression analyses were conducted to compare expression levels within patients and between the indicated timepoints: BL = baseline, TP1 = week 1, TP2 = week 2, TP8 = week 8 after pharmacotherapy; e.g., TP1vs BL indicates those genes differentially expressed between TP1 as compared to BL. The number of genes differentially expressed at a threshold of p < 0.005 between each pair of time points are indicated. Those genes showing a change in expression across multiple comparisons are indicated in the intersections of each diagram. In **(A)**, the number of genes showing increased expression is indicated in red, while the number of genes showing decreased expression is indicated in green. The total number of differentially expressed genes is shown in **(B)**.

The comparison of baseline to week 1, 2, and 8 samples revealed 156, 121, and 585 differentially expressed probes, respectively, at an uncorrected threshold of *p* < 0.005 (**Figure 1**). We did not explore differences between groups treated with ESC *versus* BUP given the small sample sizes when stratifying by treatment. GE data therefore were collapsed across treatments to examine the effects of treatment on GE irrespective of the specific drug used.

Only a subset of gene transcripts showed a change in expression at more than one time point, with 7 shared between the first and second weeks, 31 between the first and eighth week, 14 between the second and eighth week, and only 4 across first-, second-, and eight-week time points. To limit the number of comparisons in further data analyses, we restricted our detailed investigations to the most robust effect of those transcripts showing differential expression: 8 weeks compared to baseline (585 genes).

An examination of GO.BP categories revealed increased expression for several process terms, although none of these terms were statistically significant at the 0.05 level, after correction for multiple comparisons with FDR. The top 10 GO.BP process terms are shown in **Table 2A**. To supplement enrichment analyses of GO terms, we also tested for enrichment of gene pathways using a compiled set of lists from CHDI. While there was increased expression of several of these pathways, none were significantly enriched after correction for multiple comparisons (FDR *p* > 0.05). The top 10 pathway terms are presented in **Table 2B**.

**Table 2A:** Enrichment of top 10 gene ontology biological process terms in the global treatment effect gene set.

Gene Ontology biological process terms (N genes overlapping)	FDR corrected *q*-value
Translational termination (14)	0.46
Viral life cycle (21)	0.64
Cellular metabolic process (244)	0.64
Cellular component disassembly (31)	0.64
Cellular protein complex disassembly (16)	0.64
Nuclear-transcribed mRNA catabolic process (13)	0.64
mRNA metabolic process (27)	0.64
Translational elongation (13)	0.64
mRNA catabolic process (13)	0.64
Submandibular salivary gland formation (2)	0.64

**Table 2B:** Enrichment of top 10 gene pathways in the global treatment effect gene set.

Gene pathways (N genes overlapping)	FDR corrected *q*-value
Reactome pathway: influenza viral RNA transcription and replication (10)	0.08
Reactome pathway: eukaryotic translation and elongation (9)	0.08
Reactome pathway: influenza life cycle (11)	0.08
Reactome pathway: influenza infection (11)	0.08
Reactome pathway: translation (11)	0.08
Reactome pathway: GTP hydrolysis and joining of the 60S ribosomal subunit (9)	0.08
Reactome pathway: peptide chain elongation (8)	0.08
Reactome pathway: eukaryotic translation and termination (8)	0.08
Reactome pathway: viral mRNA translation (8)	0.08
Reactome pathway: SRP-dependent co-translational protein targeting to membrane (9)	0.08

To further interrogate these results, we submitted the list of 585 differentially expressed genes (at uncorrected p < 0.05) for annotation enrichment to DAVID (at an uncorrected threshold of p < 0.05). Five biological process terms were significantly enriched: regulation of cell proliferation, GE, cellular biosynthetic process, positive regulation of cell proliferation, and positive regulation of biological process. Two molecular function terms were significantly enriched, specifically RNA binding and cis–trans isomerase activity.

Examination of gene sets preferentially enriched for specific cell types within the nervous system did not reveal a significant overlap between our global treatment effect gene list and genes preferentially expressed in any of these cell types.

Examination of the results of nine previous peripheral GE studies of patients undergoing antidepressant treatment and with published gene lists available for comparison revealed a significant overlap of 18 genes (FDR q = 0.046) between our global treatment effect gene list and one other published gene list (Guilloux et al., 2015) comparing non-remitters to remitters at pretreatment baseline. The list of overlapping genes is provided in **Table 3**. There also was not a significant overlap with genes expressed in the any region of the thalamus, but there was a trend (p < 0.07) toward overlap with genes involved in the Wnt/β-catenin pathway in the anterior thalamus (**Table 4**). There were a total of 10 individual genes that were identified in the present study and at least two previous studies. This list is shown in **Table 5**.

**Table 3:** Eighteen genes overlapping between treatment-related gene list and Guilloux et al., 2015.

Gene symbol	
ABCC3	ATP-binding cassette, sub-family C (CFTR/MRP), member 3
ADAR	Adenosine deaminase, RNA-specific
ADIPOR1	Adiponectin receptor 1
EPOR	Erythropoietin receptor
FAM136A	Family with sequence similarity 136, member A
GSR	Glutathione reductase
LSM7	LSM7 homolog, U6 small nuclear RNA and MRNA degradation associated
MARCKS	Myristoylated alanine-rich protein kinase C substrate
NDUFB2	NADH dehydrogenase (ubiquinone) 1 beta subcomplex, 2, 8kDa
PBX1	Pre-B-cell leukemia homeobox 1
R3HDM4	R3H domain containing 4
RASSF7	Ras association (RalGDS/AF-6) domain family (N-terminal) member 7
ROMO1	Reactive oxygen species modulator 1
RPL27	Ribosomal protein L27
RPS10	Ribosomal protein S10
SP2	Sp2 transcription factor
SUGP2	SURP and G-patch domain-containing 2
TMEM208	Transmembrane protein 208

**Table 4:** Thirteen genes overlapping between treatment-related gene list and genes selected by Wisniewska et al. (2012).

Gene symbol	
DPYSL5	Dihydropyrimidinase-like 5
FGF10	Fibroblast growth factor 10
GABRG2	Gamma-aminobutyric acid (GABA) A receptor, gamma 2
HOXC6	Homeobox C6
ITGA7	Integrin, alpha 7
KDM2B	Lysine (K)-specific demethylase 2B
MARCKS	Myristoylated alanine-rich protein kinase C substrate
QRFP	Pyroglutamylated RFamide peptide
RAP2B	RAP2B, member Of RAS oncogene family
SDPR	Serum deprivation response
SMYD2	SET and MYND domain-containing 2
SYT5	Synaptotagmin V
TPM1	Tropomyosin 1 (alpha)

**Table 5:** Genes showing differential expression in current study and at least two previous studies.

Gene name	Number of previous studies showing differential expression	Study 1	Study 2	Study 3	Study 4	Gene function
map2k3	2	Belzeaux2012suppletable.txt	Pettai et al., 2016 mmc1.cvs			Signaling pathway (dual specificity kinase)
sp2	3	Belzeaux2012suppletable.txt	Belzeaux et al., 2016 mmc3.csv	Guilloux et al., 2015		Transcriptional factor (activates mRNA synthesis)
atxn10	2	Belzeaux2012suppletable.txt	Mamdani et al., 2011 tp201112x1.csv tp201112x2.csv			Signaling pathway (activates the Ras-MAP kinase pathway)
chchd1	2	Belzeaux et al., 2016 mmc3.csv	Mamdani et al., 2011 tp201112x1.csv tp201112x3.csv			Mitochondrial translational factor
serac1	2	Belzeaux et al., 2016 mmc3.csv	Mamdani et al., 2011 tp201112x1.csv tp201112x3.csv			Mitochondrial phospholipid remodeling
c6orf106	2	Belzeaux et al., 2016 mmc3.csv	Mamdani et al., 2011 tp201112x1.csv tp201112x2.csv			Brain function unknown (expression associated with poor human cancer prognosis)
kdm2b	2	Belzeaux et al., 2016 mmc3.csv	Wisniewska et al., 2012 1471-2164-13-635-S1.xls			Wnt/β-catenin pathway in thalamus Regulation of histone methylation
smyd2	2	Belzeaux et al., 2016 mmc3.csv	Wisniewska et al., 2012 1471-2164-13-635-S1.xls			Wnt/β-catenin pathway in thalamus Regulation of histone methylation
MARCH8	2	Hodgson et al., 2016 Table S1	Mamdani el al., 2011 2011-12-16203-Supplementary_table4.xls			Ubiquitin protein ligase
MARCKS	4	Mamdani et al., 2011 tp201112x2.csv	Wisniewska et al., 2012 1471-2164-13-635-S1.xls	Redei et al., 2014.pdf	Guilloux et al., 2015	Wnt/β-catenin pathway in thalamus Signaling (most prominent substrate for protein kinase C)

There was no significant overlap between our gene list and previous preclinical studies (above) of drug-specific genomic signatures in the mouse brain examining the effects of either BUP or fluoxetine.

## Discussion

The results reported here are consistent with those of previous studies and indicate a significant effect of antidepressant medication treatment on GE in peripheral blood. We identified significant transcriptional changes in genes that were up- or down-regulated over the course of 8 weeks of antidepressant treatment with either ESC or BUP, two medications in widespread clinical use (American Psychiatric Association, 2010). The number of genes showing differential expression increased over the course of treatment, with 585 genes showing transcriptional changes by week 8. This emphasizes the importance of considering GE not just at end of treatment but at a series of time points during antidepressant treatment, ranging from the first week through the end of a full course of 8 weeks. There was limited overlap in the genes that were up- or down-regulated at each time point, suggesting that there may be changes in the specific pattern of gene regulation at each time point. In contrast to some previous studies, there was no relationship between changes in GE and clinical outcomes of antidepressant treatment, which may reflect heterogeneity among subjects, so that a change in expression in a particular subset of genes is sufficient to lead to clinical improvement in some individuals but not others.

The purpose of this study was to generate hypotheses and to identify genes to examine using bioinformatics tools. By comparing GE at weeks 1, 2, and 8 to baseline, we obtained 156, 121, and 585 significant differences in expression using an uncorrected threshold of *p* < 0.005. We selected this threshold to be in line with previous recommendations for preventing bias in this type of analysis (Carvalho et al., 2016). However, there is no convention for selecting an appropriate statistical threshold in genome-wide GE analyses of such samples, because the preprocessing and analysis steps tend to vary widely across groups. While it is common to use a threshold of p < 0.05 with a fold-change greater than 1.5, for example, this is arbitrary. Moreover, the use of 0.05 as the threshold in our study would likely have identified a very large number of genes and included potentially spurious findings. Our use of a threshold of p < 0.005 was motivated partly by our previous experience with similar datasets, and also reasoning that this threshold, while more conservative than p < 0.05, would not restrict the lists of significant genes to a number too small to allow further interrogation with bioinformatics tools.

This dataset is among of the largest of a growing number of studies that have examined GE in peripheral blood associated with antidepressant treatment (Mamdani et al., 2011; Belzeaux et al., 2012; Mamdani et al., 2014; Redei et al., 2014; Guilloux et al., 2015; Hodgson et al., 2016; Belzeaux et al., 2016; Jansen et al., 2016; Pettai et al., 2016). In contrast to several of these studies, we did not detect a significant association between changes in GE and clinical outcome (Mamdani et al., 2011; Mamdani et al., 2014; Redei et al., 2014; Belzeaux et al., 2016; Hodgson et al., 2016; Pettai et al., 2016) or between exposure to different antidepressant medications (Hodgson et al., 2016). It is possible that a larger sample size would be needed to detect these types of differences in GE. It also is possible that the therapeutic effects of antidepressants are not reflected by global GE, or that such changes are not reliably or consistently reflected in peripheral blood cells (as opposed to nervous system tissue). The use of peripheral samples is currently standard in the field and ethical and technical factors prevent ready access to nervous system cell types, except for cadaveric tissue or nasal epithelium (as recently reported for subjects with schizophrenia and autism) (Lepagnol-Bestel et al., 2008; Dong et al., 2012; Abdolmaleky et al., 2014; Choi et al., 2014; Gandal et al., 2018). This approach is less likely to be useful for studies of MDD or the response to antidepressants; while MDD is a chronic and recurrent condition, the phenotype of illness is not always expressed consistently, and response to antidepressant frequently is time-limited.

A subset of the 585 genes showing differential expression at 8 weeks showed similar changes at week 1 (31) and week 2 (14) comparisons with baseline (**Figure 1**). The differences in GE over time suggests that there may be different phases of gene regulation over the course of antidepressant treatment, with some genes showing only early transcription changes and fewer showing consistent change over time. GE studies of peripheral blood would be particularly well-suited to detect such transient state-dependent changes. It also is possible, however, that a number of the changes detected at only one or two time points during treatment represent false positive changes in GE. Future studies should examine changes in GE over time to verify the present finding.

To characterize the nature of genes identified in our resulting global-treatment-effect gene list, we used a number of data-driven tools, which allowed us to test whether there was an overlap between our gene list and reference gene list(s) greater than expected by chance. Our analyses using GO terms or pathways did not show a significant overlap; by contrast, annotation enrichment to DAVID revealed an enrichment of terms related to several basic biological processes (e.g., GE, cellular biosynthetic process) as well as cell proliferation. This finding is consistent with the observation that antidepressant drugs can affect a variety of different cellular processes (Blier et al., 1998; Miller et al., 2009; Sharp, 2012; Duhr et al., 2014; Stokes et al., 2015; Sun et al., 2015; Björkholm and Monteggia, 2016).

We did not detect an enrichment for any genes that were preferentially expressed in nervous system cell types. The lack of a detectable signal for genes enriched in neurons may at first pass be surprising, because the putative targets of ESC and BUP are neuronally expressed monoamine transporters. The clinical response to these drugs, however, is unlikely to be confined to the particular receptor to which the drugs initially bind. Rather, the long-term, therapeutic response is likely to involve neurons in pathways that are downstream from the initial drug target (Duman et al., 2016). It is also possible that the lack of a signal associated with neuronal or glial cell types may reflect the limitations of using a non-neuronal cell type for our genomic analysis. Peripheral blood cells provide a convenient source for human mRNA and are a common tissue source from living patients. It is likely that some transcriptional pathways are shared between white blood cells and neuronal tissue, but it is even more likely that pathways would be shared with microglia, as both function as elements of the immune system.

We did detect a significant overlap between the genes differentially expressed in the present study and one previous study (Guilloux et al., 2015). These genes involve a variety of cellular processes, most frequently involving gene regulation and transcription (**Table 3**). This finding is consistent with a putative role for antidepressant medications in increasing neuroplasticity through changes in GE.

We also detected a trend toward an overlap with genes that are differentially expressed in the Wnt/β-catenin pathway in the anterior thalamus (**Table 4**). Wnt signaling is increasingly recognized as playing an important role in the mature central nervous system, and abnormalities in this signaling pathway have been implicated in MDD (Voleti and Duman, 2012; Zhou et al., 2016; Tayyab et al., 2018). Nuclear β-catenin accumulates in mature neurons throughout the nervous system, particularly anterior thalamic cells where it plays a role in regulating expression of voltage- and ligand-gated ion channels (Wisniewska et al., 2012). Although the precise anatomic site(s) of antidepressant activity are not known, the anterior thalamus has the highest concentrations of serotonin transporter receptor sites in the brain and has been postulated to be a primary site of action of SSRI antidepressant medications such as ESC. Multiple reports have shown changes in cerebral oscillatory activity in prefrontal region during antidepressant treatment on a time frame similar to the GE changes reported here (Cook et al., 2002; Cook et al., 2005; Bruder et al., 2008; Bares et al., 2010; Leuchter et al., 2015; Leuchter et al., 2017).

There were 10 genes showing differential expression in the current study that showed differential expression in at least two previous treatment studies or in the Wnt/β-catenin pathway (**Table 5**). Two of these genes are involved in cell signaling pathways, four in gene regulation, three in the Wnt/β-catenin pathway, and one of unknown function. The fact that Wnt/β-catenin pathway genes show expression changes in multiple studies is intriguing because changes in prefrontal rhythmic oscillatory activity have been reported as a reproducible specific biomarker of ESC efficacy. It is tempting to speculate that genes regulating excitability such as voltage- and ligand-gated ion channels in some brain regions might be differentially expressed in patients treated with antidepressants. This possibility remains speculative but may be relevant to GWAS studies linking loci near several channel genes to several psychiatric disorders.

We did not examine GE changes in patients treated with ESC *versus* BUP because our sample size was likely too small to reliably detect differences in expression associated with particular antidepressants, such as have been reported in larger studies of ESC and nortriptyline (Hodgson et al., 2016). Our findings of a robust change in GE when collapsing across treatments suggest it is possible that the major initial or downstream effects of current antidepressants at a molecular level are relatively similar, despite variability in the response of particular patients to each drug (Rush et al., 2011). Despite differences in the immediate effect of antidepressants on extracellular amine concentrations, it is possible that the downstream therapeutic effects of antidepressants occur *via* common pathways. If so, the molecular signatures of divergent antidepressants could be quite similar regardless of which molecules they bind to initiate these events.

These results must be interpreted within the context of several limitations of this study. First, while this is among the larger studies of GE during antidepressant treatment, the sample size of 64 subjects limits the statistical power to detect differential expression as well as association with treatment efficacy. Second, the unusual design of the study in which treatment was changed from ESC to BUP in some subjects restricts our ability to attribute changes in expression to any particular antidepressant treatment. Nevertheless, the consistency in GE changes over time and the overlap with one relevant previous study suggests that there may be a shared signal underlying treatment response that is detectable in peripheral blood across studies. Further studies of drug-induced GE will help us to triangulate on loci that are relevant to depression. The power afforded by integrating results across additional datasets may help overcome the limited sample sizes in most extant studies. It is possible that reliable detection of significant changes will require peripheral blood samples from thousands of patients during treatment. If so, very large-scale efforts or alternative methods may be needed to address these questions.

## Ethics Statement

This study was carried out in accordance with the recommendations of the UCLA Institutional Review Board (Medical IRB 3), with written informed consent obtained from all subjects prior to any experimental procedures. All subjects gave written informed consent in accordance with the Declaration of Helsinki; our protocol was reviewed and approved by the UCLA Institutional Review Board and protected this vulnerable population of individuals with depression by means including providing them with the opportunity to review their decision about participation with family, friends, and advisors in advance.

## Author Contributions

Performed treatment study: IAC, AFL. Performed genetic analyses: SPH. Performed data analyses: IAC, EC, GC, AMH. Wrote manuscript: AFL, DEK, IAC.

## Funding

This work was supported by ARRA grant R01MH085925-02S1 (IAC: PI) and intramural resources at the University of California (AFL, SPH).

## Conflict of Interest Statement

IC discloses that he has received research support from Covidien (formerly Aspect Medical Systems), National Institutes of Health, and NeoSync, Inc.; he has been an advisor/consultant/reviewer for Arctica Health, Cerêve, HeartCloud, NeuroDetect, NeuroSigma, NIH (ITVA), U.S. Departments of Defense and Justice, and the VA (DSMB); he is editor of the Patient Management section of the American Psychiatric Association’s FOCUS journal; his biomedical intellectual property is assigned to the Regents of the University of California, and he has stock options in NeuroSigma, where he has served as Chief Medical Officer. GC discloses that he has received research funding from Takeda Pharmaceutical Company Ltd. for an unrelated project. DK discloses that he has received research support from the National Institutes of Health. He currently serves as an investigator on a study sponsored by Neosync, Inc. AL discloses that he has received research support from the National Institutes of Health, Neuronetics, Department of Defense, CHDI Foundation, and NeuroSigma, Inc. He has served as a consultant to NeoSync, Inc., Ionis Pharmaceuticals, Inc., and ElMindA. He is Chief Scientific Officer of Brain Biomarker Analytics LLC (BBA). AL owns stock options in NeoSync, Inc. and has equity interest in BBA. The remaining authors declare that the research was conducted in the absence of any commercial or financial relationships that could be construed as a potential conflict of interest.

## References

[B1] Abdolmaleky H. M. Nohesara S. Ghadirivasfi M. Lambert A. W. Ahmadkhanita H. Ozturk S. (2014). DNA hypermethylation of serotonin transporter gene promoter in drug naïve patients with schizophrenia. Schizophr. Res. 152, 373–380. 10.1016/j.schres.2013.12.007 24411530 PMC7863587

[B2] American Psychiatric Association (2010) practice guidelines for the treatment of patients with major depressive disorder. The American Psychiatric Association practice guidelines for the psychiatric evaluation of adults, https://psychiatryonline.org/pb/assets/raw/sitewide/practice_guidelines/guidelines/mdd.pdf

[B3] Bares M. Brunovsky M. Novak T. Kopecek M. Stopkova P. Sos P. (2010). The change of prefrontal QEEG theta cordance as a predictor of response to bupropion treatment in patients who had failed to respond to previous antidepressant treatments. Eur. Neuropsychopharmacol. 20, 459–466. 10.1016/j.euroneuro.2010.03.007 20421161

[B4] Bartova L. Berger A. Pezawas L. (2010). Is there a personalized medicine for mood disorders? Eur. Arch. Psychiatry Clin. Neurosci. 260, 121–126. 10.1007/s00406-010-0152-8 20957381

[B5] Baudry A. Mouillet-Richard S. Launay J. M. Kellermann O. (2011). New views on antidepressant action. Curr. Opin. Neurobiol. 21, 858–865. 10.1016/j.conb.2011.03.005 21530233

[B6] Belzeaux R. Bergon A. Jeanjean V. Loriod B. Formisano-Tréziny C. (2012). Responder and nonresponder patients exhibit different peripheral transcriptional signatures during major depressive episode. Transl. Psychiatry 2, e185. 10.1038/tp.2012.112 23149449 PMC3565773

[B7] Belzeaux R. Formisano-Tréziny C. Loundou A. Boyer L. Gabert J. Samuelian J. C. (2010). Clinical variations modulate patterns of gene expression and define blood biomarkers in major depression. J. Psychiatr. Res. 44, 1205–1213. 10.1016/j.jpsychires.2010.04.011 20471034

[B8] Belzeaux R. Lin C. W. Ding Y. Bergon A. Ibrahim E. C. Turecki G. (2016). Predisposition to treatment response in major depressive episode: a peripheral blood gene coexpression network analysis. J. Psychiatr. Res. 81, 119–126. 10.1016/j.jpsychires.2016.07.009 27438688

[B9] Bianchi M. T. Botzolakis E. J. (2010). Targeting ligand-gated ion channels in neurology and psychiatry: is pharmacological promiscuity an obstacle or an opportunity? BMC Pharmacol. 10, 3. 10.1186/1471-2210-10-3 20196850 PMC2838756

[B10] Björkholm C. Monteggia L. M. (2016). BDNF—a key transducer of antidepressant effects. Neuropharmacology 102, 72–79. 10.1016/j.neuropharm.2015.10.034 26519901 PMC4763983

[B11] Blier P. Piñeyro G. Mansari M. Bergeron R. de Montigny C. (1998). Role of somatodendritic 5-HT autoreceptors in modulating 5-HT neurotransmission. Ann. N Y Acad. Sci. 861, 204–216. 10.1111/j.1749-6632.1998.tb10192.x 9928258

[B12] Bruder G. E. Sedoruk J. P. Stewart J. W. McGrath P. H. Quitkin F. M. Tenke C. E. (2008). Electroencephalographic alpha measures predict therapeutic response to a selective serotonin reuptake inhibitor antidepressant: pre-and post-treatment findings. Biol. Psychiatry 63, 1171–1177. 10.1016/j.biopsych.2007.10.009 18061147 PMC2652474

[B13] Carvalho A. F. Köhler C. A. Fernandes B. S. Quevedo J. Miskowiak K. W. Brunoni A. R. (2016). Bias in emerging biomarkers for bipolar disorder. Psychol. Med. 46, 2287–2297. 10.1017/S0033291716000957 27193198

[B14] Choi J. Ababon M. R. Soliman M. Lin Y. Brzustowicz L. M. Matteson P. G. (2014). Autism associated gene, engrailed2, and flanking gene levels are altered in post-mortem cerebellum. PLoS One 9, e87208. 10.1371/journal.pone.0087208 24520327 PMC3919719

[B15] Cook I. A. Hunter A. M. Gilmer W. S. Iosifescu D. V. Zisook S. Burgoyne K. S. (2013). Quantitative electroencephalogram biomarkers for predicting likelihood and speed of achieving sustained remission in major depression. J. Clin. Psychiatry 74, 51–56. 10.4088/JCP.10m06813 23419226

[B16] Cook I. A. Leuchter A. F. Morgan M. Witte E. Stubbeman W. F. Abrams M. (2002). Early changes in prefrontal activity characterize clinical responders to antidepressants. Neuropsychopharmacology 27, 120–131. 10.1016/S0893-133X(02)00294-4 12062912

[B17] Cook I. A. Leuchter A. F. Morgan M. L. Stubberman W. Siegman B. Abrams M. (2005). Changes in prefrontal activity characterize clinical response in SSRI nonresponders: a pilot study. J. Psychiatr. Res. 39, 461–466. 10.1016/j.jpsychires.2004.12.002 15992554

[B18] Dong E. Gavin D. P. Chen Y. Davis J. (2012). Upregulation of TET1 and downregulation of APOBEC3A and APOBEC3C in the parietal cortex of psychotic patients. Transl. Psychiatry 2, e159. 10.1038/tp.2012.86 22948384 PMC3565208

[B19] Duhr F. Déléris P. Raynaud F. Séveno M. Morisset-Lopez S., Mannoury la Cour C. (2014). Cdk5 induces constitutive activation of 5-HT6 receptors to promote neurite growth. Nat. Chem. Biol. 10, 590–597. 10.1038/nchembio.1547 24880860

[B20] Duman R. S. Aghajanian G. K. Sanacora G. Krystal J. H. (2016). Synaptic plasticity and depression: new insights from stress and rapid-acting antidepressants. Nat. Med. 22, 238–249. 10.1038/nm.4050 26937618 PMC5405628

[B21] Gandal M. J. Haney J. R. Parikshak N. N. Leppa V. Ramaswami G. Harti C. (2018). Shared molecular neuropathology across major psychiatric disorders parallels polygenic overlap. Science 359, 693–697. 10.1126/science.aad6469 29439242 PMC5898828

[B22] Guilloux J. P. Bassi S. Ding Y. Walsh C. Turecki G. Tseng G. (2015). Testing the predictive value of peripheral gene expression for nonremission following citalopram treatment for major depression. Neuropsychopharmacology 40, 701–710. 10.1038/npp.2014.226 25176167 PMC4289958

[B23] Hamilton M. (1960). A rating scale for depression. J. Neurol. Neurosurg. Psychiatry 23, 56–62. 10.1136/jnnp.23.1.56 14399272 PMC495331

[B24] Hawrylycz M. J. Lein E. S. Guillozet-Bongaarts A. L. Shen E. H. Ng L. Miller J. A. (2012). An anatomically comprehensive atlas of the adult human brain transcriptome. Nature 489, 391. 10.1038/nature11405 22996553 PMC4243026

[B25] Hennings J. M. Uhr M. Klengel T. Weber P. Pütz B. Touma C. (2015). RNA expression profiling in depressed patients suggests retinoid-related orphan receptor alpha as a biomarker for antidepressant response. Transl. Psychiatry 5, e538. 10.1038/tp.2015.9 25826113 PMC4429173

[B26] Hodgson K. Tansey K. E. Powell T. R. Coppola G. Uher R. Zvezdana Dernovšek M. (2016). Transcriptomics and the mechanisms of antidepressant efficacy. Eur. Neuropsychopharmacol. 26, 105–112. 10.1016/j.euroneuro.2015.10.009 26621261

[B27] Jansen R. Penninx B. W. Madar V. Xia K. Milaneschi Y. Hottenga J. J. (2016). Gene expression in major depressive disorder. Mol. Psychiatry 21, 339–347. 10.1038/mp.2015.57 26008736

[B28] Langfelder P. Horvath S. (2008). WGCNA: an R package for weighted correlation network analysis. BMC Bioinf. 12, 559. 10.1186/1471-2105-9-559 PMC2631488 19114008

[B29] Lepagnol-Bestel A. M. Maussion G. Boda B. Cardona A. Iwayama Y. Delezoide A. L. (2008). SLC25A12 expression is associated with neurite outgrowth and is upregulated in the prefrontal cortex of autistic subjects. Mol. Psychiatry 13, 385–397. 10.1038/sj.mp.4002120 18180767

[B30] Leuchter A. F. Cook I. A. Gilmer W. S. Marangell L. B. Burgoyne K. S. Howland R. H. (2009a). Effectiveness of a quantitative electroencephalographic biomarker for predicting differential response or remission with escitalopram and bupropion in major depressive disorder. Psychiatry Res. 169, 132–138. 10.1016/j.psychres.2009.04.004 19709754

[B31] Leuchter A. F. Cook I. A. Marangell L. B. Gilmer W. S. Burgoyne K. S. Howland R. H. (2009b). Comparative effectiveness of biomarkers and clinical indicators for predicting outcomes of SSRI treatment in major depressive disorder: results of the BRITE-MD study. Psychiatry Res. 169, 124–131. 10.1016/j.psychres.2009.06.004 19712979

[B32] Leuchter A. F. Hunter A. M. Jain F. A. Tartter M. Crump C. Cook I. A. (2017). Escitalopram but not placebo modulates brain rhythmic oscillatory activity in the first week of treatment of major depressive disorder. J. Psychiatr. Res. 84, 174–183. 10.1016/j.jpsychires.2016.10.002 27770740

[B33] Leuchter A. F. Hunter A. M. Krantz D. E. Cook I. A. (2015). Rhythms and blues: modulation of oscillatory synchrony and the mechanism of action of antidepressant treatments. Ann. N Y Acad. Sci. 1344, 78–91. 10.1111/nyas.12742 25809789 PMC4412810

[B34] Liu Z. Li X. Sun N. Xu Y. Meng Y. Yang C. (2014). Microarray profiling and co-expression network analysis of circulating lncRNAs and mRNAs associated with major depressive disorder. PLoS One 9, e93388. 10.1371/journal.pone.0093388 24676134 PMC3968145

[B35] Mamdani F. Berlim M. T. Beaulieu M. M. Labbe A. Merette C. Turecki G. (2011). Gene expression biomarkers of response to citalopram treatment in major depressive disorder. Transl. Psychiatry 1, e13. 10.1038/tp.2011.12 22832429 PMC3309465

[B36] Mamdani F. Berlim M. T. Beaulieu M. M. Turecki G. (2014). Pharmacogenomic predictors of citalopram treatment outcome in major depressive disorder. World J. Biol. Psychiatry 15, 135–144. 10.3109/15622975.2013.766762 23530732 PMC5293541

[B37] Menke A. Arloth J. Pütz B. Weber P. Klengel T. Mehta D. (2012). Dexamethasone stimulated gene expression in peripheral blood is a sensitive marker for glucocorticoid receptor resistance in depressed patients. Neuropsychopharmacol. 37, 1455–1464. 10.1038/npp.2011.331 PMC3327850 22237309

[B38] Miller A. H. Maletic V. Raison C. L. (2009). Inflammation and its discontents: the role of cytokines in the pathophysiology of major depression. Biol. Psychiatry 65, 732–741. 10.1016/j.biopsych.2008.11.029 19150053 PMC2680424

[B39] Miller J. A. Cai C. Langerfelder P. Geschwind D. H. Kurian S. M., Salomon, Dr, (2011). Strategies for aggregating gene expression data: the collapseRows R function. BMC Bioinf. 12, 322. 10.1186/1471-2105-12-322 PMC3166942 21816037

[B40] Miyata S. Kurachi M. Okano Y. Sakurai N. Kobayashi A. Harada K. (2016). Blood transcriptomic markers in patients with late-onset major depressive disorder. PloS One 11, e0150262. 10.1371/journal.pone.0150262 26926397 PMC4771207

[B41] Mullins N. Hodgson K. Tansey K. E. Perroud N. Majer W. Mors O. (2014). Investigation of blood mRNA biomarkers for suicidality in an independent sample. Transl. Psychiatry 4, e474. 10.1038/tp.2014.112 25350297 PMC4350518

[B42] Pettai K. Milani L. Tammiste A. Võsa U. Kolde R. Eller T. (2016). Whole-genome expression analysis reveals genes associated with treatment response to escitalopram in major depression. Eur. Neuropsychopharmacol. 26, 1475–1483. 10.1016/j.euroneuro.2016.06.007 27461515

[B43] Redei E. E. Andrus B. M. Kwasny M. J. Seok J. Cai X. Ho J. (2014). Blood transcriptomic biomarkers in adult primary care patients with major depressive disorder undergoing cognitive behavioral therapy. Transl. Psychiatry 4, e442–e442. 10.1038/tp.2014.66 25226551 PMC4198533

[B44] Rush A. J. Trivedi M.H. Stewart J.W. Nierenberg A.A. Fava M. Kurian B.T. (2011). Combining medications to enhance depression outcomes (CO-MED): acute and long-term outcomes of a single-blind randomized study. Am. J. Psychiatry 168, 689–701. 10.1176/appi.ajp.2011.10111645 21536692

[B45] Rush A. J. Trivedi M. H. Ibrahim H. M. Carmody T. J. Arnow B. Klein D. N. (2003). The 16-Item quick inventory of depressive symptomatology (QIDS), clinician rating (QIDS-C), and self-report (QIDS-SR): a psychometric evaluation in patients with chronic major depression. Biol. Psychiatry 54, 573–583. 10.1016/S0006-3223(02)01866-8 12946886

[B46] Sharp T. (2012). Molecular and cellular mechanisms of antidepressant action. Behav. Neurobiol. Depress. Treat. Curr. Top. Behav. Neurosci. 14, 309–325. 10.1007/7854_2012_216 22865463

[B47] Sheehan D. V. Lecrubier Y. Seehan K. H. Armorim P. Janavs J. Weiller E. (1998). The Mini-International Neuropsychiatric Interview (M.I.N.I.): the development and validation of a structured diagnostic psychiatric interview for DSM-IV and ICD-10. J. Clin. Psychiatry 20, 22–33, https://www.psychiatrist.com/jcp/article/Pages/1998/v59s20/v59s2005.aspx 9881538

[B48] Stokes L. Spencer S. J. Jenkins T. A. (2015). Understanding the role of P2X7 in affective disorders—are glial cells the major players? Front. Cell. Neurosci. 9, 258. 10.3389/fncel.2015.00258 26217184 PMC4495333

[B49] Sun H. Damez-Werno D. M. Scobie K. N. Shao N. Y. Dias C. Rabkin J. (2015). ACF chromatin-remodeling complex mediates stress-induced depressive-like behavior. Nat. Med. 21, 1146. 10.1038/nm.3939 26390241 PMC4598281

[B50] Tayyab M. Shahi M. H. Farheen S. Mariyath M. P. M. Khanam N. Castresana J. S. (2018). Sonic hedgehog, Wnt, and brain-derived neurotrophic factor cell signaling pathway crosstalk: potential therapy for depression. J. Neurosci. Res. 96, 53–62. 10.1002/jnr.24104 28631844

[B51] Tylee D. S. Kawaguchi D. M. Glatt S. J. (2013). On the outside, looking in: a review and evaluation of the comparability of blood and brain “-omes.”. Am. J. Med. Genet. 162, 595–603. 10.1002/ajmg.b.32150 24132893

[B52] Voleti B. Duman R. S. (2012). The roles of neurotrophic factor and Wnt signaling in depression. Clin. Pharmacol. Ther. 91, 333–338. 10.1038/clpt.2011.296 22205198

[B53] Watanabe S. Y. Iga J. Ishil K. Numata S. Shimodera S. Fujita H. (2015). Biological tests for major depressive disorder that involve leukocyte gene expression assays. J. Psychiatr. Res. 66, 1–6. 10.1016/j.jpsychires.2015.03.004 25943949

[B54] Wisniewska M. B. Nagalski A. Dabrowski M. Misztal K. Kuznicki J. (2012). Novel β-catenin target genes identified in thalamic neurons encode modulators of neuronal excitability. BMC Genomics 13, 635. 10.1186/1471-2164-13-635 23157480 PMC3532193

[B55] Zhang Y. Chen K. Sloan S. A. Bennett M. L. Scholze A. R., O’Keeffee, (2014). An RNA-sequencing transcriptome and splicing database of glia, neurons, and vascular cells of the cerebral cortex. J. Neurosci. 34, 11929–11947. 10.1523/JNEUROSCI 25186741 PMC4152602

[B56] Zhou W. J. Xu N. Kong L. Sun S. C. Xu X. F. Jia M. Z. (2016). The antidepressant roles of Wnt2 and Wnt3 in stress-induced depression-like behaviors. Transl. Psychiatry 6, e892. 10.1038/tp.2016.122 27622936 PMC5048193

